# Evaluation of the In Vivo Skin Penetration of TXVector by Confocal Raman Spectroscopy

**DOI:** 10.1111/jocd.16696

**Published:** 2024-11-27

**Authors:** Daniel Winn, Andrea Gilreath, David Pajuelo, Justyna M. Meissner, Jose L. Mullor

**Affiliations:** ^1^ Actera Ingredients Inc Newtown Pennsylvania USA; ^2^ Bionos Biotech S.L., LabAnalysis Life Science, S.r.L. Biopolo La Fe, IIS La Fe Valencia Spain

**Keywords:** hyperpigmentation, in vivo, Raman spectroscopy, skin penetration, tranexamic acid

## Abstract

**Background:**

Tranexamic acid (TXA) is a promising active to treat hyperpigmentation disorders, such as melasma and acne scars. However, TXA is highly hydrophilic and its penetration into the skin is poor and formulation dependent.

**Aims:**

In this study, our aim was to evaluate the in vivo skin penetration of an ester‐modified TXA, TXVector, directly on the skin of volunteers.

**Methods:**

For the analysis of in vivo skin penetration of TXVector, we used in vivo confocal Raman spectroscopy (CRS). The use of CRS on live skin allows us to study directly how a compound affects skin composition at different depths and how this compound penetrates into the skin in real time.

**Results:**

Our results showed that the TXA absorption into the skin via TXVector was 2.1‐fold and 3.8‐fold higher compared to free TXA 3% and TXA 1% formulations, respectively. Most importantly, upon application of TXVector, the TXA penetration flux into the skin was 107% and 280% higher than that of the free TXA 3% and TXA 1% formulations, respectively.

**Conclusions:**

In summary, this study shows that the esterification‐based TXVector formulation enhances the penetration flux of TXA and increases its bioavailability in the skin.

## 1 Introduction

The use of tranexamic acid (TXA) in the dermatology industry has recently gained attention for its efficacy in treating a number of skin disorders [1]. TXA is a synthetic derivative of the amino acid lysine, and its water solubility allows it to be easily formulated into different types of dosage forms, such as oral tablets, injectables, and topical solutions.

One of the main applications of TXA is the treatment of melasma, a common, acquired, chronic hyperpigmentation disorder characterized by light to dark brown spots on the face, typically appearing on the forehead, nose, and cheeks [2]. It has been shown that topical treatment with TXA reduces the severity of melasma, and even with a higher patient satisfaction than the gold‐standard treatment for this condition, hydroquinone [3, 4, 5, 6, 7, 8, 9]. In addition, different studies showed that TXA is effective against post‐inflammatory hyperpigmentation and erythema, common pigmentary disorders that occur as a complication of severe acne. In this regard, TXA treatment is able to reduce acne, papules, pustules, skin redness, scaling, and erythema, given its anti‐inflammatory properties [10, 11, 12]. Topical treatment with TXA has also been proved to be effective against the chronic inflammatory skin disease rosacea, by restoring the skin barrier function known to be associated with this skin condition [13, 14, 15].

The mechanism of action in topically applied TXA for melasma has been related to the suppression of cytokine/chemical mediator production. These mediators stimulate melanin production via the keratinocyte‐derived urokinase‐type plasminogen activator and plasminogen derived from dermal blood vessels in the basal layer of the epidermis, therefore inhibiting the excess of melanin production [16]. However, although the depigmentation activity of TXA is well established, its skin penetration is poor given the strong hydrophilic nature of its chemical structure, being a challenge for its topical application. This is the reason why the skin penetration of TXA is formulation‐dependent and requires a delivery system that facilitates its penetration across the hydrophobic environment of the upper layers of the skin [17].

Esterification can enhance the lipophilicity of a drug or active compound, making it more permeable through the lipid‐rich outer layer of the skin, the stratum corneum. This increased lipophilicity facilitates the drug's ability to penetrate the skin barrier more effectively [18, 19, 20, 21]. Additionally, once the esterified drug penetrates the skin, esterases present in the skin can hydrolyze the ester bond, releasing the active drug within the deeper layers of the skin. This strategy is often used in prodrug design to improve the topical delivery and efficacy of dermatological treatments.

**FIGURE 1:**
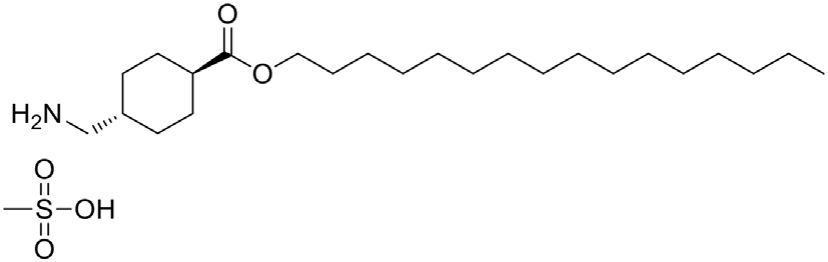
Representation of the molecular structure of TXVector.

In vivo confocal Raman spectroscopy is a non‐invasive technique that analyzes the chemical composition of living tissues at a molecular level. In vivo confocal Raman spectroscopy applied to the dermatology field is a powerful tool not only to study the chemical composition of the skin of volunteers, but also to analyze the penetration of drugs or active compounds into the skin in an accurate, informative, and non‐invasive manner [22]. In this study, we evaluated the in vivo skin penetration of a novel lipophilic prodrug based on an ester modification of TXA (TXVector) by in vivo confocal Raman spectroscopy (Figure 1). Previous studies have shown that the ester conjugate of TXA provides clinical depigmentation benefits at relatively low use levels [23]. In this study, we aimed to evaluate whether this clinical benefit may be due to enhanced skin permeability provided by the lipophilicity of TXVector. Our results showed that TXVector increased the amount of TXA delivered into the skin and its penetration flux (speed of penetration) compared to free TXA formulations, demonstrating that TXVector is an efficient system to facilitate penetration of TXA into the skin.

## 2 Materials and Methods

### 2.1 Study Subjects

A total of 10 subjects were enrolled in the study according to the following inclusion criteria: (i) males or females between 20 and 45 years old, (ii) who were in good general health (physical, mental, and social well‐being, not merely the absence of disease/infirmity). The established exclusion criteria were: (i) allergy or reactivity to some of the components of the products, (ii) recent surgery in the experimental area, (iii) relevant cutaneous marks in the experimental areas which could interfere with the measurements (scars, sunburns, etc.), (iv) in‐use relevant pharmacological or hormonal treatment, (v) presence of skin diseases or melanomas, (vi) forecast of change of routine or relevant way of life during the period of study, and (vii) nursing, pregnant, or planning to become pregnant during the study. The detailed information about the study subjects is described in Table S1.

### 2.2 Institutional Review Board Statement and Informed Consent

This clinical study was conducted at the facilities of Bionos Biotech S.L. The study was conducted according to the guidelines of the Declaration of Helsinki, and approved by the Institutional Review Board (or Ethics Committee) of Bionos Biotech S.L. (Date 28/08/2023 and Code number 075‐2023). The study protocol, including the inclusion/exclusion criteria, is in accordance with the Scientific Committee on Consumer Safety (SCCS) guidance. It meets all international standards for research studies involving human subjects, the Good Clinical Practices (ICH‐GCP), and World Medical Association. Informed consent was obtained from each volunteer prior to initiating the study describing reasons for the study, possible adverse effects, associated risks and potential benefits of the treatment, and their limits of liability. The panelists signed and dated the informed consent document to indicate his authorization to proceed and acknowledge his understanding of the contents before the start of the study.

### 2.3 Treatment of Volunteers

For the evaluation of the product penetration, the 10 selected study subjects were enrolled in a one‐day topical treatment. In this study, three different formulations were tested for their penetration capacity into the skin: (i) tranexamic acid (TXA) at 1% in water (hereafter TXA 1%), (ii) TXA at 3% in water (hereafter TXA 3%), and (iii) an ester‐modified TXA at 3% in water (hereafter TXVector). TXA was purchased from Sigma Aldrich, while TXVector (trade name of the cetyl tranexamate mesylate molecule) was acquired from Actera Ingredients [23]. Based on the structure of the TXVector molecule, a 3% solution of this compound is equivalent to a dose of free TXA at 1%. First, three square areas of 4 cm^2^ each were delimited in different areas of the volar forearm of each subject, one corresponding to each one of the tested formulations (TXA 1%, TXA 3% or TXVector). 50 μL of each product were applied with a sterilized pipette in the corresponding delimited area, and were gently and evenly spread. The chemical composition of the skin was analyzed in the delimited areas before the application of the formulations, and 1, 10, and 20 min after the application.

### 2.4 Assessment of the Product Penetration Into the Skin

The measurements of the chemical composition of the skin were based on the confocal Raman spectroscopy technology, by using the Gen2 SCA Ultimate (RiverD International B.V., Rotterdam, Netherlands). This instrument is a confocal Raman system of high sensitivity designed for in vivo skin analysis [24, 25]. The gen2‐SCA Ultimate has two built‐in lasers (wave class 3B lasers, 671 nm and 785 nm). For this measurement, the laser with a wavelength of 785 nm was used, recording the “Raman fingerprint region” with wavenumbers from 400 to 1800 cm^−1^. The laser power complies with the maximum permissible levels for skin as defined by the international laser safety standard (IEC 60285–1:2007; < 30 mW for 785 nm, and < 20 mW for 671 nm). In the 400–1800 cm^−1^ region, spectra of the skin were acquired at 5‐μm increments in the axial direction up to a depth of 15 μm using a 5‐s acquisition time per point and a pinhole of 50 μm. Concentration profiles of the tested products in the stratum corneum relative to the amount of skin protein were obtained with the SkinTools software (RiverD International B.V., Rotterdam, Netherlands) using a previously reported fitting algorithm [26]. Briefly, the algorithm consists of a least square fitting of the Raman spectra obtained in vivo to a library of Raman spectra of stratum corneum molecular components obtained in vitro, resulting in a set of fit coefficients for the stratum corneum constituent spectra. The fit coefficients are subsequently normalized to the fit coefficient for the protein spectrum to compensate for the loss of signal intensity for increasing skin depths. In order to specifically quantify the amount of TXA and the ester‐modified TXA (TXVector) in the skin measurements, the Raman spectra of both molecules were acquired in vitro with the gen2‐SCA Ultimate and included in the library of Raman spectra used by the SkinTools software, to subsequently obtain the concentration profiles of TXA and TXVector. Data were expressed as μg TXA or TXA‐ester per mg of protein, and μg TXA or TXA‐ester per cm^2^ of stratum corneum (assuming a stratum corneum depth of 15 μm); for the representation of the penetration flux, the data were represented in μg of compound per cm^2^ of stratum corneum*h.

### 2.5 Statistical Analysis

Data were statistically analyzed with the Graphpad Prism software by the one‐way ANOVA test and Dunnet's T3 post hoc multiple comparisons test. Statistical significance was declared at *p* < 0.05, 95% of confidence. Bars in the charts represent the mean value for each condition, and error bars indicate the standard error of the mean (SEM) for each group of values.

## 3 Results

### 3.1 Tranexamic Acid Penetration Into the Skin of Volunteers

TXA is a molecule with a poor penetration capacity through the lipid‐rich stratum corneum layer given its highly hydrophilic nature. In this study, the TXA molecule was modified with an ester group in order to increase its penetration into the skin. The rationale of this modification lies on the fact that the ester moiety allows to diffuse through hydrophobic environments and it is subsequently cleaved and released by cellular esterases, hence releasing the bounded molecule (TXA). For this reason, we quantified two types of molecules in the skin upon the application of the tested formulations, the TXA and the TXA‐ester; the TXA molecule was quantified upon application of all three tested formulations, while the TXA‐ester molecule was only quantified upon the application of the TXVector formulation.

**FIGURE 2:**
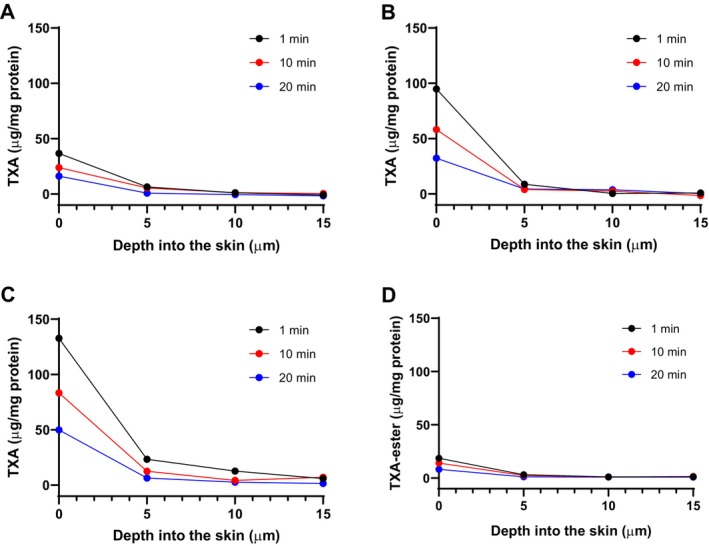
Graphical representation of the amount of TXA or TXA‐ester as a function of depth for the indicated time points. (A) TXA quantification after application of TXA 1%. (B) TXA quantification after application of TXA 3%. (C) TXA quantification after application of TXVector. (D) TXA‐ester quantification after application of TXVector. Units are expressed as μg of compound per mg of protein.

**FIGURE 3:**
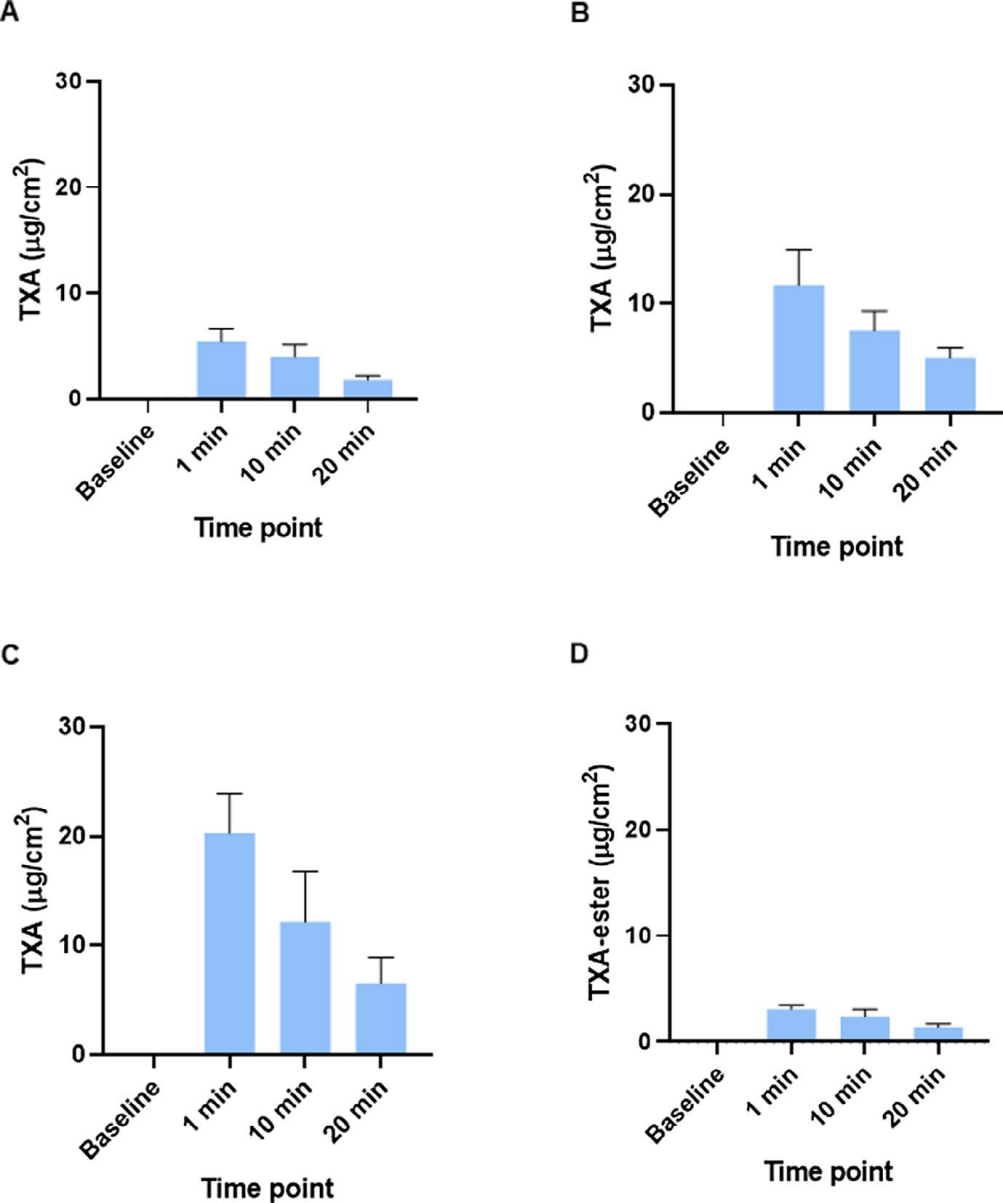
Graphical representation of the μg of TXA or TXA‐ester per cm^2^ of stratum corneum at different time points. (A) TXA quantification after application of TXA 1%. (B) TXA quantification after application of TXA 3%. (C) TXA quantification after application of TXVector. (D) TXA‐ester quantification after application of TXVector. Units are expressed as μg of compound per cm^2^ of stratum corneum. The mean ± SEM are shown.

Confocal Raman spectroscopy allows the quantification of specific molecules in different layers of the skin in vivo. The amount of the TXA and TXA‐ester molecules in the selected time points as a function of depth is shown in Figure 2, expressed as μg of compound per mg of protein. The area under the curves of the data represented in Figure 2 was analyzed and used to calculate the total amount of TXA and TXA‐ester present in the stratum corneum in vivo, expressed as μg of TXA or TXA‐ester per cm^2^ of stratum corneum of the skin at the indicated time points (Figure 3). In general, the data dispersion obtained and observed in our results is within the range normally observed for studies using in vivo confocal Raman spectroscopy [22, 27]. Our results showed that, after application of the formulations, the amount of TXA or TXA‐ester measured in subsequent time points decreases gradually in a time‐dependent manner due (i) to the diffusion of molecule to deeper layers, and/or (ii) to the metabolism or degradation of the molecule (Figures 2 and 3). We observed that the amount of TXA detected in the stratum corneum was higher after the application of TXA 3% and TXVector than after application of TXA 1%, in accordance with the amount of TXA present in these formulations (Figures 2 and 3). Interestingly, when TXVector was applied into the skin, most of the detected TXA was the free TXA, rather than the TXA‐ester (Figures 2 and 3); this observation suggests that TXVector is cleaved by esterases during the penetration into the skin.

**FIGURE 4:**
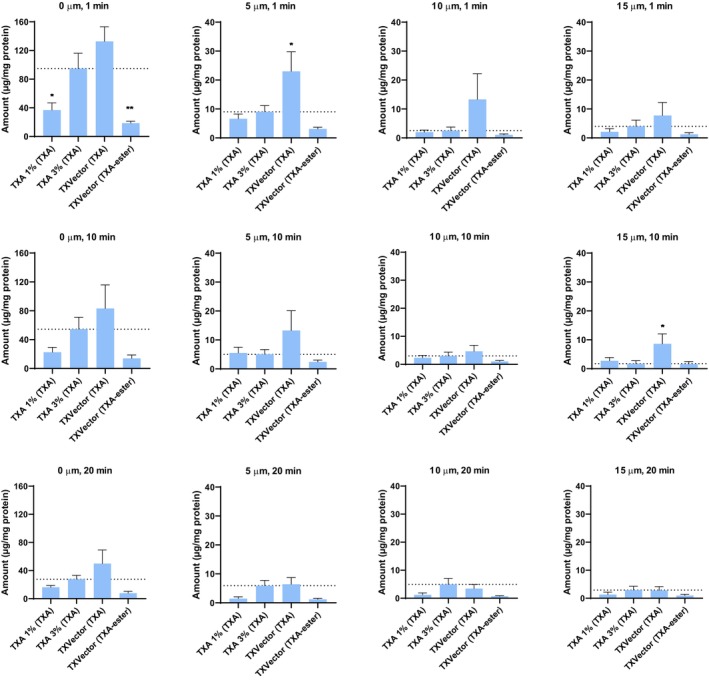
Graphical representation of the amount of TXA or TXA‐ester at specific depths and time points. The levels of TXA were evaluated for the TXA 1%, TXA 3% and TXVector formulations; the levels of TXA‐ester were evaluated for the TXVector formulation. Units are expressed as μg of compound per mg of protein. Asterisks indicate statistical significance when compared to the TXA 3% formulation. Statistical significance is indicated by asterisks and depicted as follows: *p‐value < 0.05, **p‐value < 0.01.

A more detailed analysis of the skin penetration can be made comparing separately the amount of TXA and TXA‐ester at each selected depth in all the evaluated time points, to have a more specific picture of how these molecules penetrate into the skin in vivo upon the application of the tested formulations. In Figure 4, bar graphs show the amount of TXA or TXA‐ester at specific depths and time points, after topical application of the tested formulations. In this figure, we observe that after applying TXVector, a higher amount of TXA is detected after 1 min (at 5 μm depth) and 10 min (at 15 μm depth), compared to TXA 3%. This observation is in accordance with the results of Figures 2 and 3 showing that the TXA‐ester of TXVector is digested by cellular esterases, thus releasing free TXA into the skin.

**FIGURE 5:**
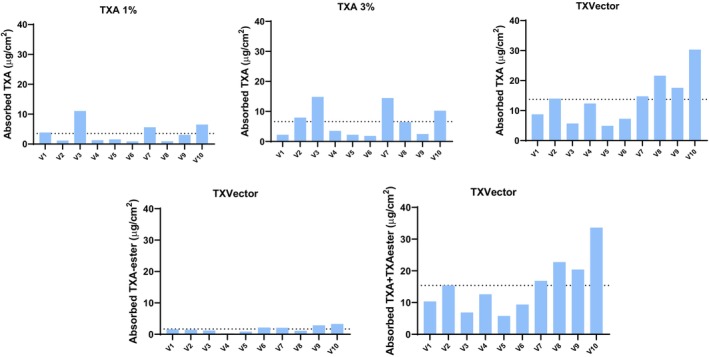
Graphical representation of the amount of absorbed TXA and/or TXA‐ester during the 20 min lapse for all volunteers separately, after application of the indicated formulations. Units are expressed as μg of compound per cm^2^ of stratum corneum. The dashed line indicates the averaged value.

In order to represent the data in a more intuitive manner, we calculated the amount of TXA or TXA‐ester absorbed into the skin per each volunteer separately. For this, the amount of compound detected in the stratum corneum at the 20 min time point was subtracted to that detected at the 1‐min time point (Figure 5). In this regard, our results show that the average amount of TXA absorbed into the skin is higher after the application of TXVector, compared to when TXA 3% or TXA 1% is applied (Figure 5).

**FIGURE 6:**
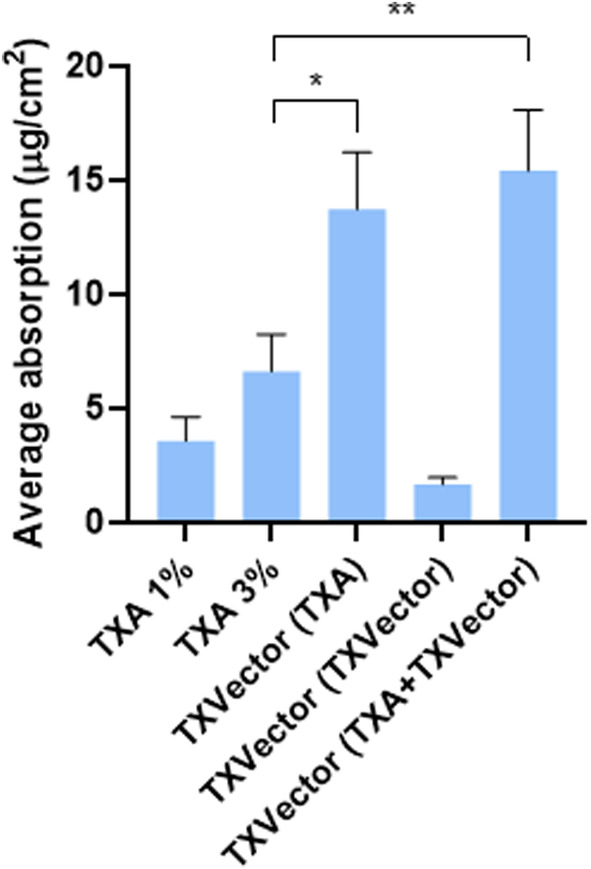
Graphical representation of the averaged amount of absorbed TXA or TXA‐ester during the 20 min lapse for all volunteers collectively, after application of the indicated formulations. Units are expressed as μg of compound per cm^2^ of stratum corneum. Statistical significance is indicated by asterisks and depicted as follows: *p‐value < 0.05, **p‐value < 0.01.

For comparative purposes, the averaged compound absorption for all volunteers is represented in Figure 6. As this figure shows, when the TXVector formulation was applied into the skin, the amount of TXA absorbed was 2.1‐fold higher than that observed for TXA 3% (p‐value < 0.05) (Figure 6). Moreover, when compared to TXA 1%, the amount of TXA absorbed was 3.8‐fold higher upon topical application of TXVector (*p*‐value < 0.05) (Figure 6).

### 3.2 Tranexamic Acid Penetration Flux

**FIGURE 7:**
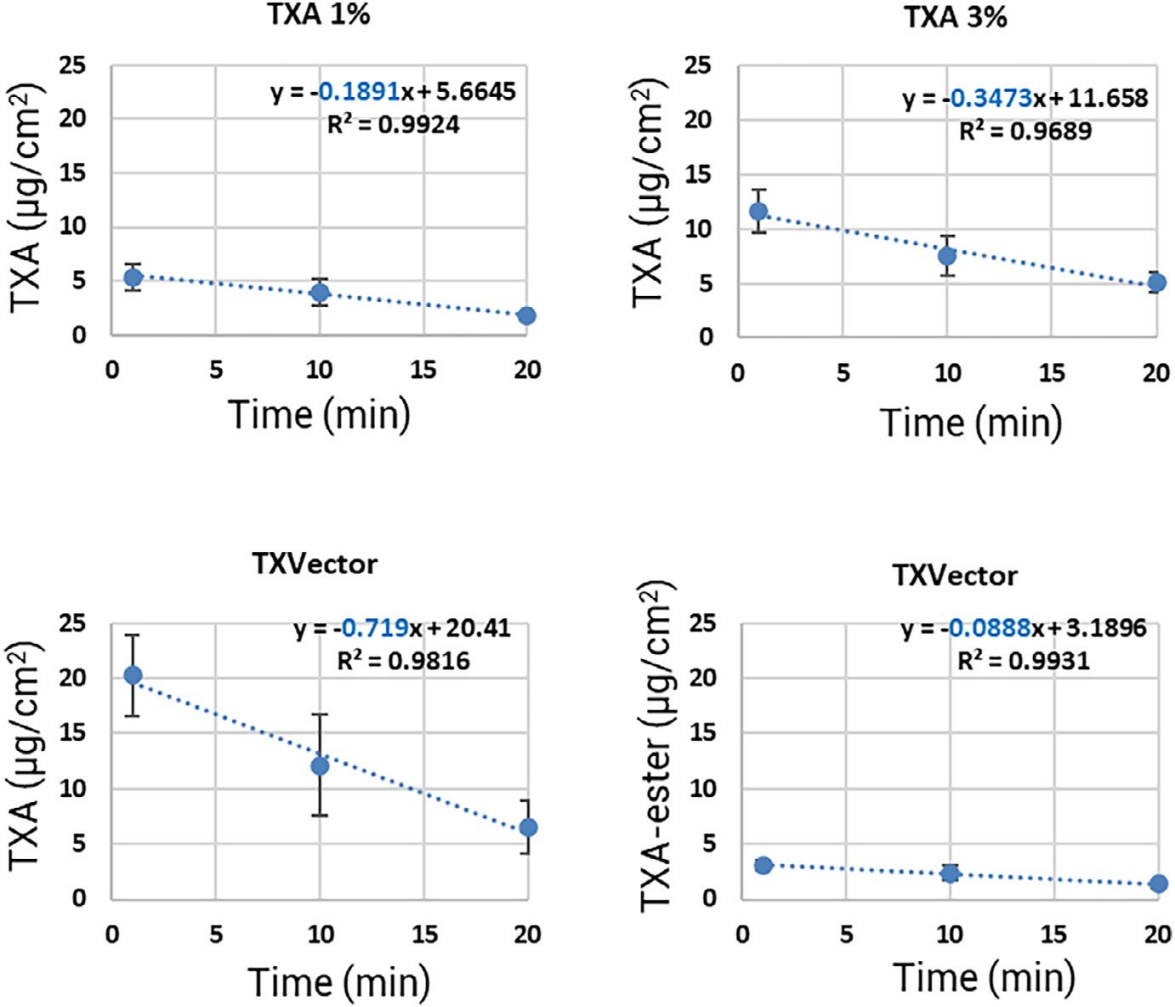
Graphical representation of the μg of TXA or TXA‐ester per cm^2^ of stratum corneum at the indicated time points. The equation, *R*‐squared and the slope values (penetration flux, in blue) are indicated.

In the dermatology field, it is of a great interest to design systems that deliver active compounds into the skin. Given the highly hydrophobic environment of the lipidic stratum corneum layer, some active compounds require a carrier system not only to get delivered to a certain depth, but also to penetrate into the skin at a higher speed. In this regard, we calculated the penetration flux of TXA after the application of the three tested formulations of this study, where the penetration flux refers to the μg of compound that penetrate per cm^2^ of skin per unit of time. The graphs of Figure 7 represent the amount of TXA or TXA‐ester (μg per cm^2^ of stratum corneum) at the indicated time points. In the graphical representations, data points are connected by a straight dash line. The equation and R‐squared of those lines were calculated and indicated in each graph. The value of the slope in each equation is marked in blue and corresponds to the penetration flux of the quantified compound into the skin in that time interval. Table 1 represents the penetration flux of TXA or TXA‐ester expressed as μg per cm^2^ of stratum corneum*h for the tested formulations, showing that the TXA penetration flux is 11.3, 20.8 and 43.1 μg/cm^2^*h for TXA 1%, TXA 3% and TXVector, respectively. The analysis of the penetration flux into the skin revealed that the TXA penetration flux after applying TXVector is 107% higher than after applying TXA 3% (Figure 7, Table 1). Importantly, the TXA penetration flux after applying TXVector is 280% higher than after applying TXA 1% (Figure 7, Table 1). These results demonstrate that TXVector increases the penetration flux of TXA compared to its free, hydrophilic forms.

**TABLE 1:** Values of the TXA or TXA‐ester penetration flux (expressed as μg/cm^2^*h) for the indicated formulations.

Formulation	μg/cm^2^*h
TXA 1% (TXA)	11.3
TXA 3% (TXA)	20.8
TXVector (TXA)	43.1
TXVector (TXA ester)	5.3

## 4 Discussion

One of the current challenges in the dermatology field is to increase the bioavailability of drugs and active compounds in the skin upon topical application. This is particularly important for molecules that, due to their chemical nature, have a poor skin penetration capacity and cannot pass across the lipid‐rich stratum corneum layer. Therefore, the development of efficient delivery systems is of great interest for the dermatology industry when developing topical formulations. These delivery systems are based on different technologies such as liposomes, nanoparticles, microneedle patches, hydrogels, emulsions, and chemical modifications [28, 29, 30, 31]. This challenge is particularly important for the formulations based on TXA, since this molecule has a strong hydrophilic nature, and its penetration into the skin is dependent on the formulation of the final product. In this regard, different strategies have been used in order to increase the bioavailability of TXA upon topical application, such as microneedling [32, 33, 34, 35] or laser pretreatment [36, 37]. However, these methods are often associated with skin irritation and damage, risk of infection, pain, discomfort, dependence of trained personnel, and a higher cost. For this reason, there is a need to develop new formulations to enhance TXA bioavailability upon topical application.

In this study, we evaluated the skin penetration capacity of a novel ester‐modified form of TXA, TXVector. Given the hydrophilic nature of TXA limiting its penetration into the skin, we hypothesized that our ester modification of TXA (TXVector) improves skin permeability and TXA bioavailability. Therefore, we evaluated the skin penetration of both active principles (TXA and TXVector) without being included in any formulation to avoid the potential interference of other formulated molecules. For this, we used in vivo confocal Raman spectroscopy, a state‐of‐the‐art technology that allows the non‐invasive, quantitative analysis of skin penetration of topical formulations directly in the skin of volunteers, bypassing the limitations of ex vivo systems (Franz cell) and providing valuable information to optimize active delivery [22, 38, 39]. Confocal Raman spectroscopy measures directly the TXA molecule through the detection of its Raman emission spectrum with an unparalleled level of accuracy in vivo. This is the first study evaluating the skin penetration of a TXA formulation in vivo with confocal Raman spectroscopy.

We observed that the concentration of TXA or the ester form of TXA in the stratum corneum decreased in a time‐dependent manner. This observation suggests that these molecules are being metabolized (and therefore no longer detectable through their specific Raman spectra) and/or diffused to deeper layers of the skin, falling below the detection limit of the instrument, as previously observed in similar studies with other actives [22, 40]. In addition, and in accordance to the actual TXA concentration of each tested sample, the concentration of TXA in the skin upon application of the TXA 3% formulation was higher than that upon application of the TXA 1% formulation. This finding indicates that higher concentrations of TXA in topical formulations are more effective in increasing the bioavailability of TXA in the skin, supported by other clinical studies using TXA concentrations of 3% to 5% [8, 41, 42, 43, 44].

Esterification of active principle is a common strategy to improve the penetration through the lipid‐rich outer layer of the skin, the stratum corneum [18, 19, 20, 21]. In this study, we found that esterification of TXA enhances its lipophilicity, making it more compatible with the lipid‐rich outer layer of the skin and therefore improving the penetration of TXA into deeper layers of the skin. We observed that esterification of TXA (TXVector) to enhance its skin penetration capacity increased the amount of TXA absorbed into the skin by 2.1‐fold and 3.8‐fold compared to TXA 3% and TXA 1% formulations, respectively, demonstrating that the ester form of TXA (TXVector) is a much more skin‐permeable form of TXA that increases the bioavailability of this molecule in the skin in vivo. Most importantly, in this study we found evidences that the ester form of TXA (TXVector) is metabolized by cellular esterases during the process of penetration, therefore releasing the free form of TXA into the skin. This conclusion is based on the observations that when TXVector was applied on the skin, most of the detected TXA was free TXA rather than the TXA‐ester. This finding is in accordance with the expected molecular mechanism of the ester‐mediated transdermal penetration.

Recently, some studies described how the chemical modification of TXA improved its penetration capacity. Tang and colleagues showed that a TXA‐loaded ZIF‐8 presented a higher penetration capacity, based on the activation of the aquaporin‐3 protein (AQP‐3), and a significant improvement of the clinical signs of rosacea and melasma [45]. On the other hand, Cen and colleagues recently showed that a chemical modification of TXA with a formulation containing 2% isosorbide dimethyl ether, 1% pentanediol, and 0.5% inositol increased the skin penetration by 80% compared to free TXA in an ex vivo Franz cell‐based pig skin explant model and quantified by HPLC [46]. In this regard, the ester‐modified TXVector proved to increase the TXA penetration into the skin in a higher extent (up to 3.8‐fold), demonstrating the efficacy of TXVector in increasing TXA bioavailability in the skin. Moreover, our study provides the added value of the accuracy of the measurement by confocal Raman spectroscopy directly in the skin of volunteers, which bypasses the limitation of other techniques to study the active penetration ex vivo.

When designing an active delivery system, an important factor to consider is the penetration flux by which the active penetrates into the skin. If the active penetration flux is too slow, it might be degraded or metabolized before it reaches the skin layer where it is supposed to exert its function. Studies analyzing the raw active penetration flux by confocal Raman spectroscopy showed that values depend deeply on the active structure and formulation, ranging from 0.3 to 8 μg/cm^2^*h [22, 27, 40], while the flux for TXA upon treatment with TXVector is higher (43.1 μg/cm^2^*h). Most importantly, we found that the TXA penetration flux after applying TXVector is 107% and 280% higher than after applying TXA 3% and TXA 1%, respectively. These results demonstrate that the esterification of TXA in the TXVector formulation increases the speed by which it passes through the lipid‐rich barrier of the stratum corneum.

In conclusion, in this study we demonstrate that the esterification‐based TXVector formulation facilitates the penetration flux of TXA into the skin. Most importantly, this finding suggests that the improved clinical benefits of the TXVector in discoloration compared to free TXA may be a result of the increased bioavailability of TXA in the skin.

## Author Contributions

**Daniel Winn, Andrea Gilreath, Jose L. Mullor:** conceptualization. **Daniel Winn, Andrea Gilreath, Jose L. Mullor:** study design. **David Pajuelo:** data collection. **David Pajuelo:** data analysis. **Justyna M. Meissner:** writing original draft. **David Pajuelo, Jose L. Mullor:** validation. **David Pajuelo:** data curation. **Jose L. Mullor:** supervision. **Justyna M. Meissner, Daniel Winn, Andrea Gilreath, Jose L. Mullor:** writing, review, and editing. **Justyna M. Meissner, Daniel Winn, Andrea Gilreath, Jose L. Mullor:** revision and proofreading. All the authors read and approved the final version of the manuscript and agreed to be accountable for all aspects of the work.

## Ethics Statement

The standard protocol and test conditions were submitted to and approved by the Ethical Committee of Bionos Biotech (Date 18/07/2023 and Code number 0058‐2023).

## Conflicts of Interest

The authors declare no conflicts of interest.

## Supporting information


**Table S1.** Information of the study subjects.

## Data Availability

The data that support the findings of this study are available from the corresponding author upon reasonable request.
