# Supplementary material for: Evaluation of the In Vivo Skin Penetration of TXVector by Confocal Raman Spectroscopy
Source: J Cosmet Dermatol. 2024 Nov 27;24(2):e16696. doi: 10.1111/jocd.16696 (PMC11845972; doi:10.1111/jocd.16696)
Supplement: Supplementary file 1 — Table S1. Information of the study subjects. [file JOCD-24-e16696-s001.docx]

**Table S1**. Information of the study subjects.

| **Volunteer** | **Gender** | **Age** | **Skin type** |
| --- | --- | --- | --- |
| V1 | Female | 26 | Combination |
| V2 | Male | 40 | Dry |
| V3 | Female | 38 | Combination |
| V4 | Female | 29 | Combination |
| V5 | Male | 24 | Combination |
| V6 | Female | 32 | Dry |
| V7 | Male | 24 | Combination |
| V8 | Female | 25 | Dry |
| V9 | Male | 27 | Combination |
| V10 | Female | 33 | Combination |
